# Deciphering the mechanism of jujube vinegar on hyperlipoidemia through gut microbiome based on 16S rRNA, BugBase analysis, and the stamp analysis of KEEG

**DOI:** 10.3389/fnut.2023.1160069

**Published:** 2023-05-19

**Authors:** Guofeng Duan, Lijuan Li

**Affiliations:** ^1^College of Horticulture, Shanxi Agricultural University, Taigu, Shanxi, China; ^2^Jinzhong College of Information, Taigu, Shanxi, China

**Keywords:** jujube vinegar, high-fat diet, hyperlipoidemia, gut microbiome, BugBase

## Abstract

**Background:**

Growing data indicate that the gut microbiome may contribute to the rising incidence of hyperlipoidemia. Jujube vinegar lowers lipids, protects the liver, and reduces oxidant capacity, however, it is unknown whether this is due to the gut flora. To further research the role of the gut microbiome in treating hyperlipidemia with jujube vinegar, we looked into whether the action of jujube vinegar is related to the regulation of the gut microbiome.

**Method:**

Thirty male ICR mice were used. The control group (CON), the high-fat diet (HFD) group, and the vinegar group (VIN) each consisted of ten female ICR mice fed consistently for eight weeks. For each treatment, we kept track of body mass, liver index, blood lipid levels, and oxidative stress state. We also analyzed mouse feces using high-throughput 16srRNA sequencing to examine the relationship between jujube vinegar’s hypolipidemic effect and antioxidant activity and how it affects the gut microbiome.

**Results:**

Jujube vinegar reduced body weight by 19.92%, serum TC, TG, and LDL-C by 25.09%, 26.83%, and 11.66%, and increased HDL-C by 1.44 times, serum AST and ALT decreased by 26.36% and 34.87% respectively, the blood levels of SOD and GSH-Px increased 1.35-fold and 1.60-fold, respectively. While blood MDA decreased 33.21%, the liver’s SOD and GSH-Px increased 1.32-fold and 1.60-fold, respectively, and the liver’s MDA decreased 48.96% in HFD mice. The gut microbiome analysis revealed that jujube vinegar increased the intestinal microbial ASV count by 13.46%, and the F/B (Firmicutes/Bacteroidota) ratio by 2.08-fold in high-fat diet mice, and the proportion was significantly inversely correlated with TC, TG, and LDL-C and positively correlated with HDL-C. Biomarker bacteria in the vinegar group included *Lactobacillaceae and Lactobacillus*, which correlated favorably with HDL-C, SOD, and GSH-Px and negatively with LDL-C, TC, and TG. Jujube vinegar increased the abundance of the Aerobic, Contains Mobile Elements, and Facultative Aerobic by 2.84 times, 1.45 times, and 2.40 times, while decreased the abundance of Potential pathogens by 44.72%, according to the BugBase study. The KEGG analysis showed that jujube vinegar was predominantly reflected in the biological process of gene function and related to signal transduction pathways, including glucagon signaling system, HIF-1 signaling pathway, adipocytokine signaling pathway, amino sugar, and nucleotide sugar metabolism, and so forth.

**Conclusion:**

Based on these findings, jujube vinegar may reduce hyperlipoidemia by controlling the gut microbiome and enhancing antioxidant capacity.

## 1. Introduction

Hyperlipidemia is a metabolic disorder characterized elevated levels of total cholesterol (TC), triglyceride (TG), or low-density lipoprotein (LDL-C) and a reduction in high-density lipoprotein cholesterol (HDL-C) (1, 2). Eating foods high in triglycerides and cholesterol is linked to hyperlipidemia. It has been shown that consuming too much saturated fats from animal sources raises total serum cholesterol (TC) values and may be a factor in many illnesses (3, 4). As a result, hyperlipidemia risks human health and raises social issues. The alteration of the intestinal environment brought by a malfunction of lipid metabolism might lead to an imbalance in the microbial community (5). Diabetic(db/db) mice with lipid metabolic disorders have low levels of butyrate-producing bacteria, such as *Faecalibacterium prausnitzii*, *rectal fungi*, and *Roseburia* intestinalis, and high levels of opportunistic pathogens, such as *Clostridium Hathewayi*, *Clostridium ramosum*, and *Eggerthella* *Lenta* (5, 6). Significantly more LPS-producing and mucosa-damaging bacteria were found in the feces of rats with hyperlipidemia, such as *Bilophila*, *Sutterella*, and *Akkermansia* (7). The gut flora’s makeup will change due to a high-fat, high-sugar Western diet, which will also increase the likelihood of lipid metabolic problems (8). High-fat diet (HFD) eating can impact the permeability and integrity of intestinal epithelial cells by increasing Gram-negative bacteria and LPS levels and reducing intestinal expressions of ZO-1 and occludin, two tight junction proteins. LPS enters the bloodstream and is detected and bound by LPS-binding proteins. These proteins subsequently combine with macrophage CD14 to create the LPS-CD14 complex, which activates some intracellular processes *via* TLR-4. Inflammatory factors are expressed and released as a result, which impairs the ability of the liver, fat, and muscle to control lipid metabolism and results in diseases of lipid metabolism (9).

Vinegar is primarily classified into grain and fruit vinegar based on the raw materials used. Both types are produced by the anaerobic fermentation of saccharides into ethanol by yeast and the aerobic oxidation of ethanol to acetic acid by a particular bacterial species (10). Fruit vinegar contains melanin, polysaccharides, and other macromolecular materials and is being rich in tiny molecular organic acids, phenols, and mineral elements (11). According to studies, vinegar can lower blood cholesterol levels, inhibits oxidation, protects against liver and cardiovascular disorders, is antimicrobial, anti-tumor, and anti-aging and fruit vinegar can effectively regulate lipid metabolism and lessen liver damage in hyperlipidemic mice (12, 13). Fruit vinegar’s blood-lipid-lowering benefits can be attributed, in large part, to its antioxidant and anti-inflammatory properties (14, 15). However, because different sources of carbs and microorganisms are used during the fermentation process, the bioactivities and health benefits of vinegar may vary (16). Currently, red dates, black dates, and green dates are the primary sources of jujube vinegar (16, 17). Caffeic acid, ferulic acid, flavonoid, and carotenoid comprise the bulk of jujube vinegar’s biological functions (18, 19). *In vivo* and *in vitro* antioxidant activity is the primary biological function of jujube vinegar (17, 18, 20). This research used “wood jujube” as the raw material for making jujube vinegar, which contains phenols, flavonoids, and acids as its main bioactive substances. However, the impact of jujube vinegar on the gut flora and antioxidant capacity of hypolipemic mice, has received scant attention. In order to assess the effects of jujube vinegar on the mice given an HFD, this study looked at its hypolipidemic activity, antioxidant capacity, and impacts on the gut microbiome.

## 2. Materials and methods

### 2.1. Jujube vinegar and a high-fat diet

Jujube vinegar was produced by the Shanxi Agricultural University’s Vinegar Research Center, and we showed its ingredients in Supplementary Table 1. The high-fat diet included 45% fat from Botchy Hongdae Biotechnology Co., Ltd., No. HD001.

### 2.2. Experimental design and animals

Male ICR mice that were 8 weeks old (*n* = 30) were bought from Shanxi Medical University. The mice were kept in a cage at room temperature (22°C ± 1°C), with a 12-h dark/photoperiod and 50 ± 5 per cent relative humidity. All mice were given a seven-day acclimatization period and were then randomly assigned to one of three groups: the control group (CON), which received a conventional diet (with 6% of energy from fat) supplemented with distilled water by oral gavage at 8:00 am every day; the high-fat group (HFD), which received a high-fat diet (with 45% fat); and the vinegar group (VIN), which received an HFD diet with jujube vinegar (1 mL/kg body weight) by oral gavage at 8:00 am every. Once a week, we take the mice’s weight. The study protocol was reviewed and approved by the Shanxi Agriculture University Institutional Animal Care and Use Committee of Shanxi Agriculture University (Approval number: SXAU-EAW-2018 M002010). All experiments were performed in accordance with Institutional Guidelines on Animal Experimentation at Shanxi Agriculture University.

### 2.3. Collection and preparation of samples

After a 12-h fast, the mice were anesthetized, blood was collected through the mice’s eyeballs, and the samples were spun for 15 minutes at 4 degrees Celsius and 3,000 revolutions per minute. The resulting supernatant was then subjected to biochemical analysis.

The entire liver and abdominal fat were dissected. Liver weight was divided by total body weight to determine the liver index. The abdominal fat index was calculated by dividing the abdominal fat weight by the body weight.For SOD, GSH-Px, and MDA analysis, we froze 50 mg of liver tissue in liquid nitrogen and stored it at –20 degrees Celsius.

### 2.4. Biochemistry profile

Serum TG, TC, HDL-C, LDL-C, Superoxide dismutase (SOD), Glutathione Peroxidase (GSH-Px), and malondialdehyde (MDA) were determined using commercial kits (Nanjing Jiancheng Bioengineering Institute, Nanjing, Jiangsu, China).

The hepatic tissues of 0.5 mg were homogenized with 500 μL PBS and centrifuged at 5,000 r/min for 15 min to obtain the supernatant for SOD, GSH-Px, and MDA, and the methods of SOD, GSH-Px, and MDA using commercial kits (Nanjing Jiancheng Bioengineering Institute, Nanjing, Jiangsu, China).

### 2.5. 16S rRNA gene analysis

The 16S rRNA gene analysis was carried out in the same manner as previously described (21, 22). After extracting the entire DNA, the conserved regions designed the primers (F: ACTCCTA CGGGAGGCAGCA, R: GGACTACHVGGGTWTCTAAT). We used an known primer pair for microbial diversity to multiply the V3 + V4_b region of the 16S rRNA gene. The sequencing results have been uploaded to the NCBI Public Database.^1^ Trimmomatic v0.33 software was used to sort the Raw Reads, and CUTADAPT 1.9.1 software was used to find and eliminate the primer sequences, and the Clean Reads without primer sequences were obtained. We used the DADA2 method in qiime22020.6 for de-noising, two-terminal lines spliced, and the chimeric sequences removed to get the final valid data (Non-chimeric Reads) amplicon sequence variants (ASV) on the BMK Cloud platform, and the ASVs filter was applied to all sequences with a threshold of 0.0005%. The taxonomic information of each representative sequence was annotated using the Silva138 Database and the mother algorithm.

Based on the AVS sequence composition, the BMK Cloud software categorized the species^2^ The taxonomic tree maps of the samples were obtained based on the AVS analysis results at the phylum and genus levels. The Chao1, ACE, Shannon, and Simpson indices were utilized in the alpha diversity analysis that was conducted on the interventions. Principal Component Analysis (PCA) and Principal coordinates analysis (PCoA) were used to analyze the differences among samples, and anosim analysis can test whether there is significant difference in beta diversity between samples of different groups. At the level of taxonomic composition, the variation in species abundance between data was evaluated using a linear discriminant analysis (LDA) threshold of 4.0 and an LDA effect size (LEfSe). The Spearman correlation coefficient was used to investigate the relationship between blood parameters and microbiota abundance based on the species composition distribution. BugBase calculated phenotypic abundance based on 16S rRNA gene sequencing results. The Kyoto Encyclopedia of Genes and Genomes (KEGG) was predicted using Phylogenetic Investigation of Communities by Reconstruction of Unobserved States. Fisher’s test comparisons were performed between various groups using STAMP software analysis.

### 2.6. Statistical analysis

The study used the Tukey test to compare each group’s averages. Mean ± SEM showed all data.

## 3. Results

### 3.1. Jujube vinegar reduced body weight, liver index, and abdominal fat index in high-fat diet mice

**Figure 1:**
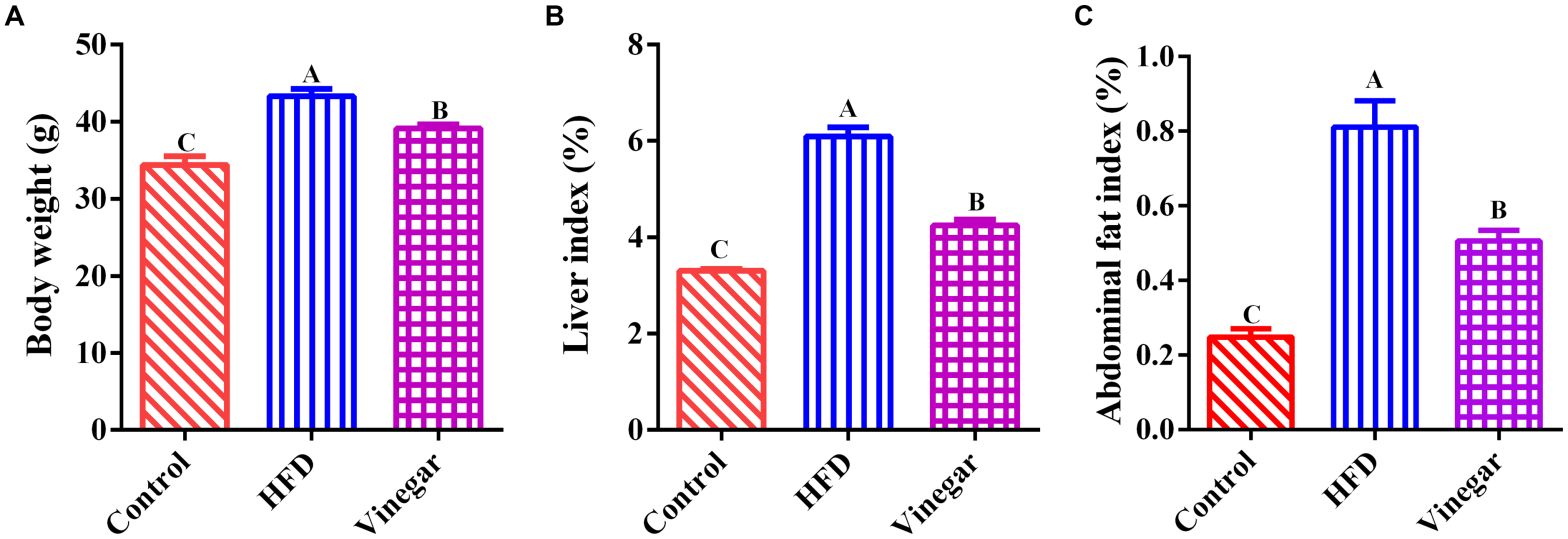
Effects of Jujube vinegar on body weight, liver index, and abdominal fat index in HFD-fed mice. **(A)** Body weight, **(B)** liver index. **(C)** abdominal fat index. Data are expressed as the means ± SEM, *n* = 10. Lowercase letters indicate significant differences (*p* < 0.05), uppercase letters indicate highly significant differences (*p* < 0.01), the same below.

The HFD and vinegar treatments had higher weights than the CON treatment (*p* < 0.01, Figure 1A) compared to the control group, The Vinegar treatment was significantly lighter weight than the HFD treatment (*p* < 0.01, Figure 1A). The HFD and VIN treatments had higher liver and abdominal fat indexes than the CON treatment; the VIN treatment had fewer liver and abdominal fat indexes than the HFD treatment (*p* < 0.01, Figures 1B,C).

### 3.2. Jujube vinegar improved blood lipid in high-fat diet mice

**Figure 2:**
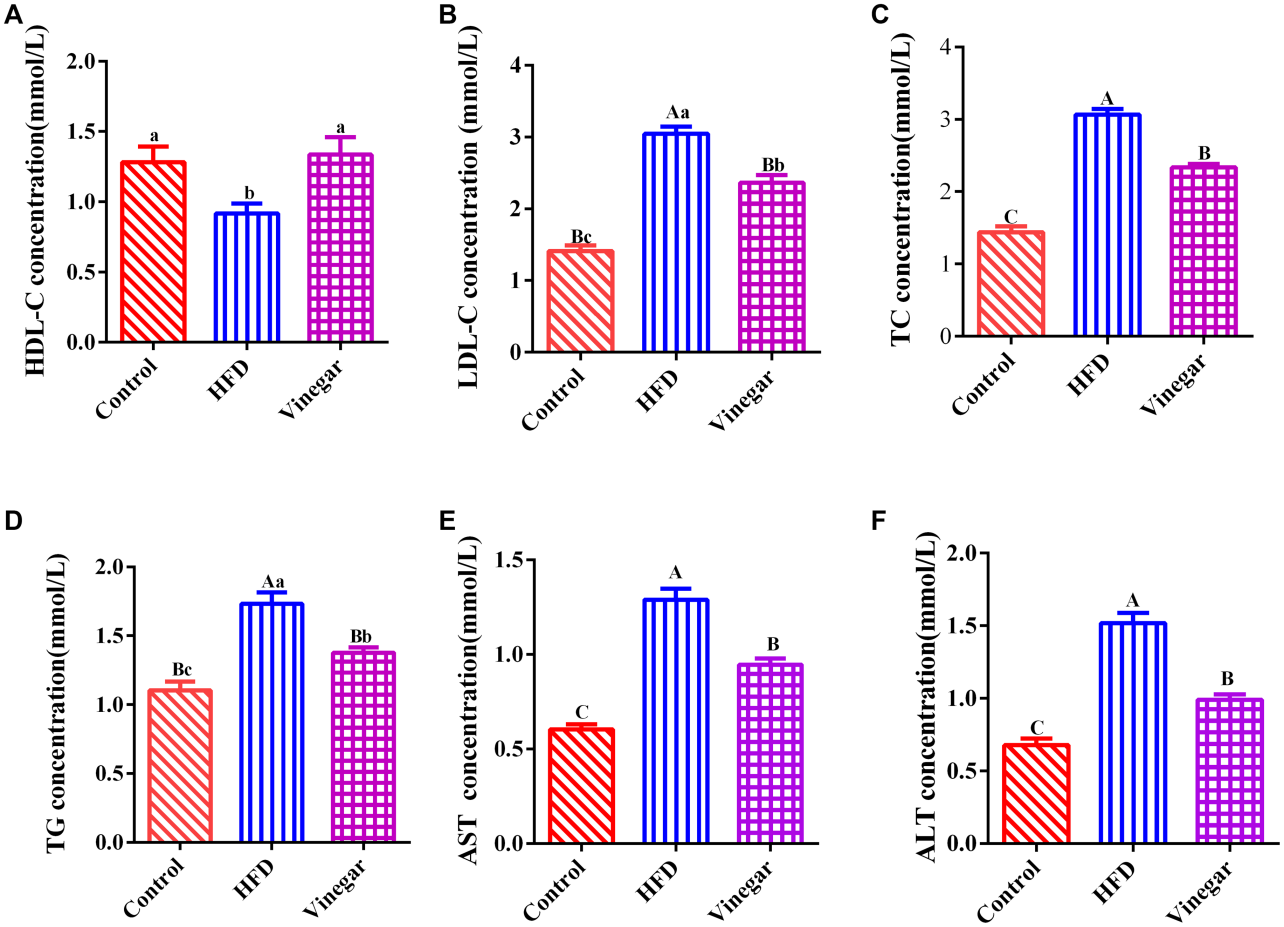
Effects of Jujube vinegar on serum lipid metabolism in HFD-fed mice. **(A)** Serum HDL-C concentration, **(B)** serum LDL-C concentration, **(C)** serum TC concentration, **(D)** serum TG concentration, **(E)** serum AST concentration, **(F)** ALT concentration. Data are expressed as the means ± SEM, *n* = 10.

Figure 2, illustrated that, in contrast to the CON treatment, the serum HDL-C concentration in the HFD treatment dropped heavily (*p* < 0.05, Figure 2A), while the serum LDL-C, TC, ALT, and AST drastically grew (*p* < 0.01, Figures 2B,C,E,F). The serum HDL-C in the VIN treatment did not differ significantly (Figure 2A). In contrast, the vinegar group’s serum LDL-C (*p* < 0.05, Figure 2B) and TC and TG fell dramatically (*p* < 0.01, Figures 2C,D). These findings implied that a hyperlipemia model could be successfully established in mice fed a diet containing 45% fat. Jujube vinegar improved liver function and reduced blood lipid levels by enhancing serum HDL-C concentration and lowering serum TC, TG, and LDL-C.

### 3.3. Jujube vinegar enhanced the antioxidant capacity in high-fat diet mice

**Figure 3:**
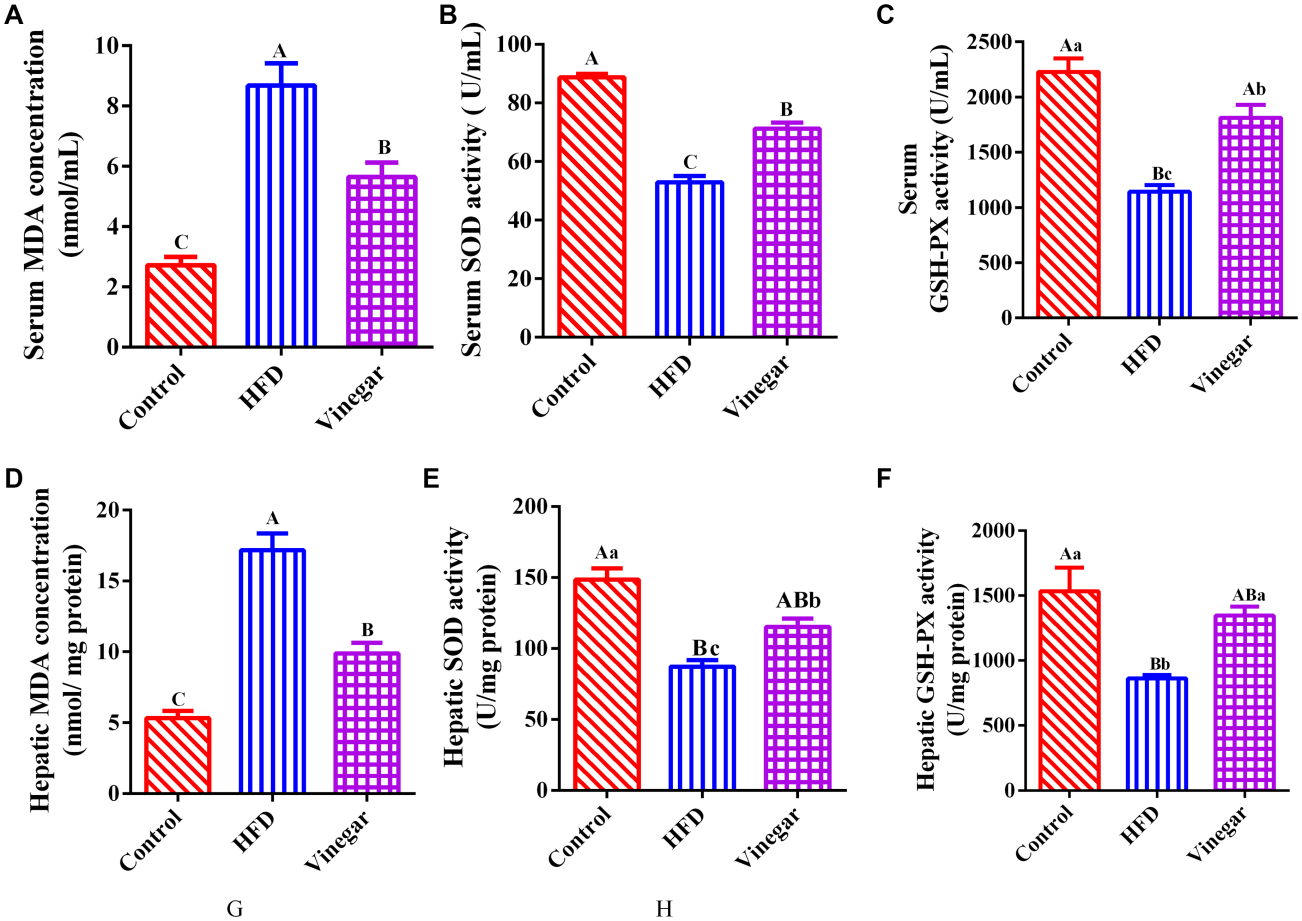
Jujube vinegar enhanced the antioxidant capacity in HFD-fed mice. **(A)** Serum MDA concentration, **(B)** serum SOD activity, **(C)** serum GSH-Px activity, **(D)** liver MDA concentration, **(E)** liver SOD activity, **(F)** liver GSH-Px activity. Data are expressed as the means ± SEM, *n* = 10.

Jujube vinegar was able to upregulate serum SOD and GSH-Px activities and downregulate serum MDA concentration in the HFD-fed mice (*p* < 0.01, Figures 3A–C). The results of hepatic SOD, GSH-Px, and MDA were broadly consistent with those of serum measurements (*p* < 0.01, Figures 3D–F). The findings showed that jujube vinegar could boost antioxidant levels in HFD mice.

### 3.4. Diversities analysis of gut microbiome in mice

**Figure 4:**
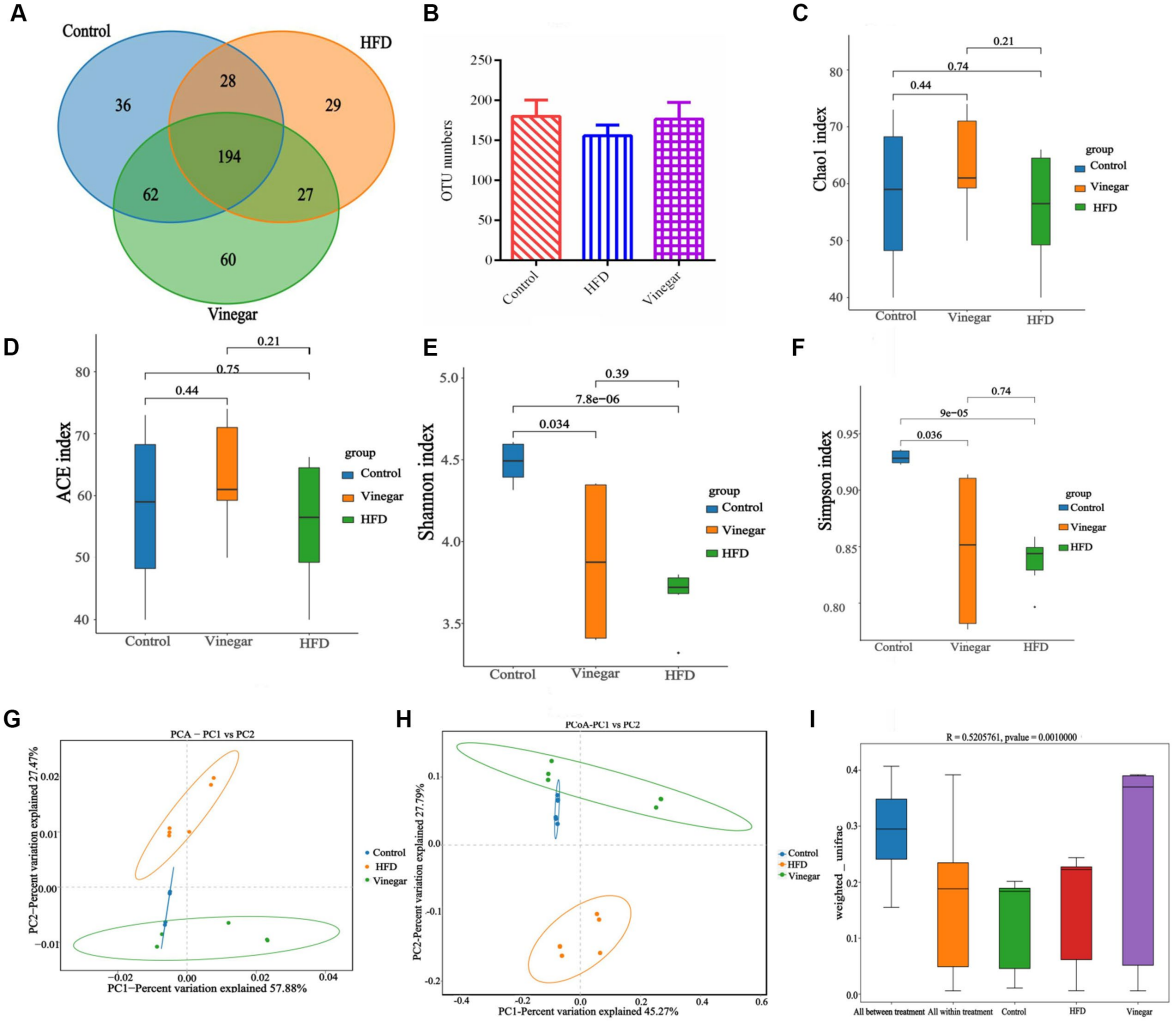
Diversities analysis of gut microbiome in mice among groups. **(A)** Venn graph, **(B)** OTU numbers, **(C)** ACE index of ɑ diversities analysis, **(D)** Chao 1 index of ɑ diversities analysis, **(E)** Shannon index of ɑ diversities analysis, **(F)** Simpson index of ɑ diversities analysis, **(G)** PCA analysis, **(H)** PCoA analyses, **(I)** Anosim analysis. Data are expressed as the means ± SEM, *n* = 6.

Sequencing yielded 1,298,475 raw reads from 18 samples, and 739,155 non-chimeric reads were produced by denoising, splicing two-terminal sequences, and deleting chimeric sequences (Supplementary Table 2). There were 436 ASVs in total (Figure 4A), of which 36 were specific to the control sample (Figure 4A), 29 ASVs to the HFD sample (Figure 4A), and 60 ASVs to the vinegar sample (Figure 4A); there were also 28 ASVs in the control and HFD treatments, 62 in the control and vinegar treatments, and 27 ASVs in the HFD and vinegar treatments (Figure 4A). The number of ASVs reduced by 13.5% in the HFD treatment compared to the CON group (Figure 4B), while it rose by 13.4% in the vinegar treatment compared to the HFD treatment (Figure 4B).

The alpha diversity of the gut bacteria was determined utilizing the Chao1, ACE, Shannon, and Simpson indices. Our results showed no statistically meaningful difference between the three treatments on the Chao1 or ACE indices, indicating that the numbers of gut microbiomes were the same in all three groups (Figures 4C,D). Figure 4E shows that the Shannon and Simpson indices were considerably less in the HFD and VIN treatments than in the CON treatment (*p* < 0.01 and *p* < 0.05). The Shannon index in the VIN treatment climbed 1.01 times more than in the HFD treatment, whereas the Simpson index declined by 3.95%. The findings demonstrated that a high-fat diet reduced the gut microbiota’s variety, evenness, and abundance of, and jujube vinegar reversed this result to some extent.

In this experiment, QIIME was used to examine the beta diversity. The results are shown in Figures 4G–I. When PC1 contribution rate was 57.88% and PC2 contribution rate was 27.44%, the results of PCA showed that the control and jujube vinegar treatments were completely separated from HFD treatment partially separated from control and jujube vinegar treatments (Figure 4G); and PC1 contribution rate was 45.27% and PC2 contribution rate was 27.79%, the results of PCoA showed that the control and jujube vinegar treatments were completely separated from HFD treatment. In contrast, the CON and VIN treatments were only partly different (Figure 4H). The results showed that the box plots of the Beta diversity between-group differences study allowed one to see the median sample similarity within the treatment intuitively, there were significant differences in the microbial community’s structure (*p* = 0.001, Figure 4I). Intestinal microbial beta diversity differed in HFD mice compared to CON and VIN treatments, remaining consistent between the two groups after jujube vinegar treatment.

### 3.5. Effects of jujube vinegar on the composition of gut microbiome in mice

**Figure 5:**
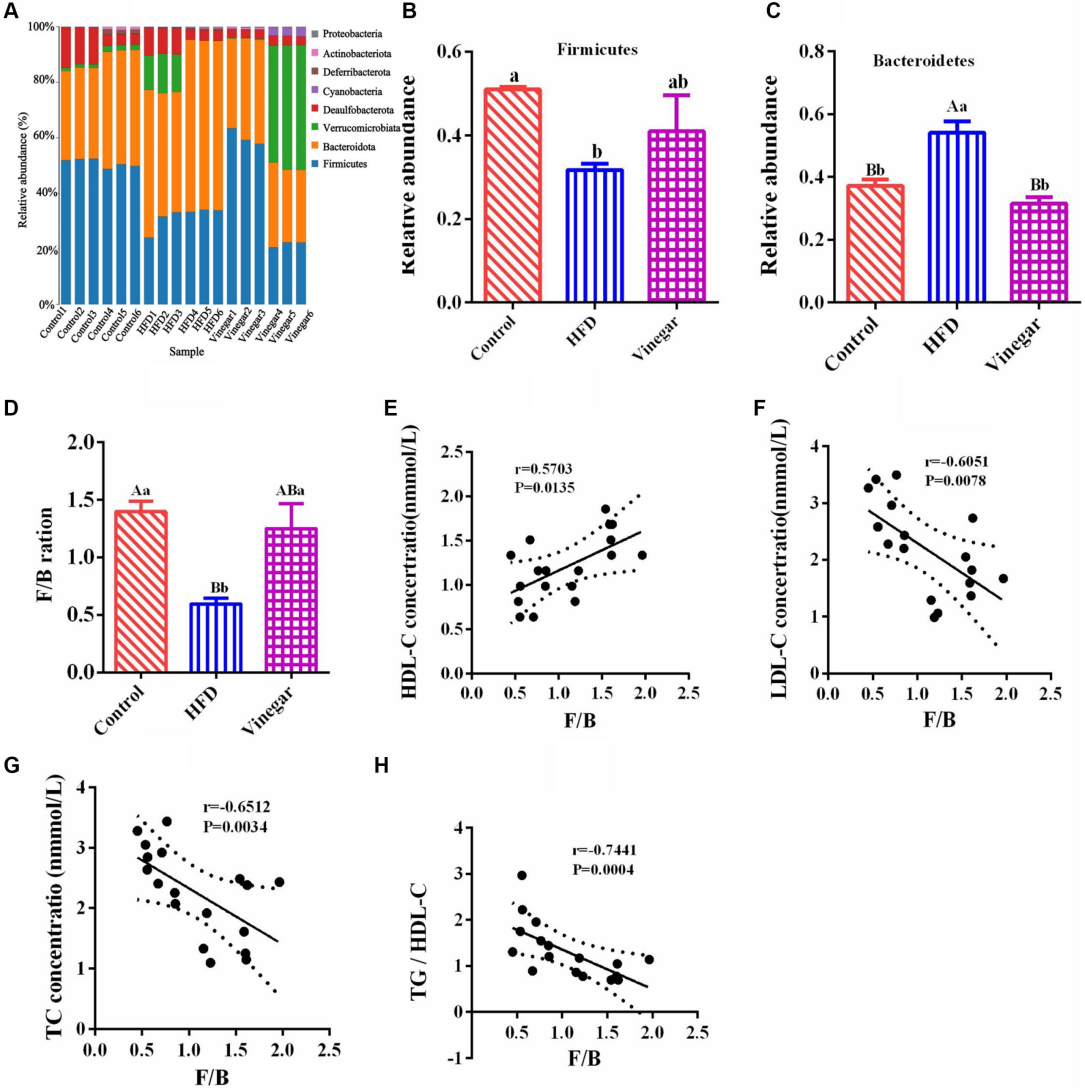
Phylum classification differences in gut microbiome among three groups. **(A)** Relative abundance distribution at phylum levels, **(B)** *Firmicutes* relative abundance, **(C)** *Bacteroidota* relative abundance, **(D)** *Firmicutes*/*Bacteroidota* (F/B) ratio, **(E)** the correlation of F/B and HDL-C, **(F)** the correlation of F/B and LDL-C, **(G)** the correlation of F/B and TC, **(H)** the correlation of F/B and TG. Data are expressed as the means ± SEM, *n* = 6.

*Bacteroidota*, *Firmicutes, Verrucomicrobiota*, *Desulfobacterota*, *Cyanobacteria*, *Deferribacterota*, *Actinobacteriota*, and *Proteobacteria* were discovered at the phylum level (Supplementary Table 3). Bacteroidetes and Firmicutes were two of the most abundant groups among them (Figure 5A). Compared with the CON group, *Firmicutes* abundance in the HFD group was dramatically declined (*p* < 0.05, Figure 5B), while *Bacteroidota* abundance in the HFD group was significantly elevated (*p* < 0.01, Figure 5C). *Firmicutes* and *Bacteroidota* abundances in the VIN treatment were not entirely different from the CON treatment (Figures 5B,C). Firmicutes in the VIN treatment raised by 21.38% more than in the HFD treatment, while Bacteroidota was substantially less abundant than in the HFD treatment (*p* < 0.01, Figure 5B). These results suggested that jujube vinegar improved the disturbance of intestinal microflora at phylum levels induced in high-fat diet mice.

The HFD group’s bar graph of *Firmicutes* to *Bacteroidota* (F/B) was considerably lower than that of the vinegar and control treatments (*p* < 0.01, Figure 5D). The F/B ratio was positively correlated with HDL-C, with correlation coefficient r = 0.5266, *p* = 0.0248 (Figure 5E); the F/B ratio is negatively correlated with LDL-C (correlation coefficient r = − 6,051, *p* = 0.0078, Figure 5F), TC (correlation coefficient r = −6,512, *p* = 0.0006, Figure 5G), and the TC/HDL-C ratio (r = − 0.7441, *p* = 0.0004, Figure 5H). These results showed that *Bacteroidota* and *Firmicutes* were the dominant bacteria in the gut. The HFD mice reduced the ratio of F/B, which caused the disorder of the prevalent bacteria in the mice’s gut. The F/B ratio was negatively correlated with TC, LDL-C, and TG/HDL-C, indicating that the lipid-lowering effect of jujube vinegar was related to the disorder of *Firmicutes* and *Bacteroidota* induced by HFD.

**Figure 6:**
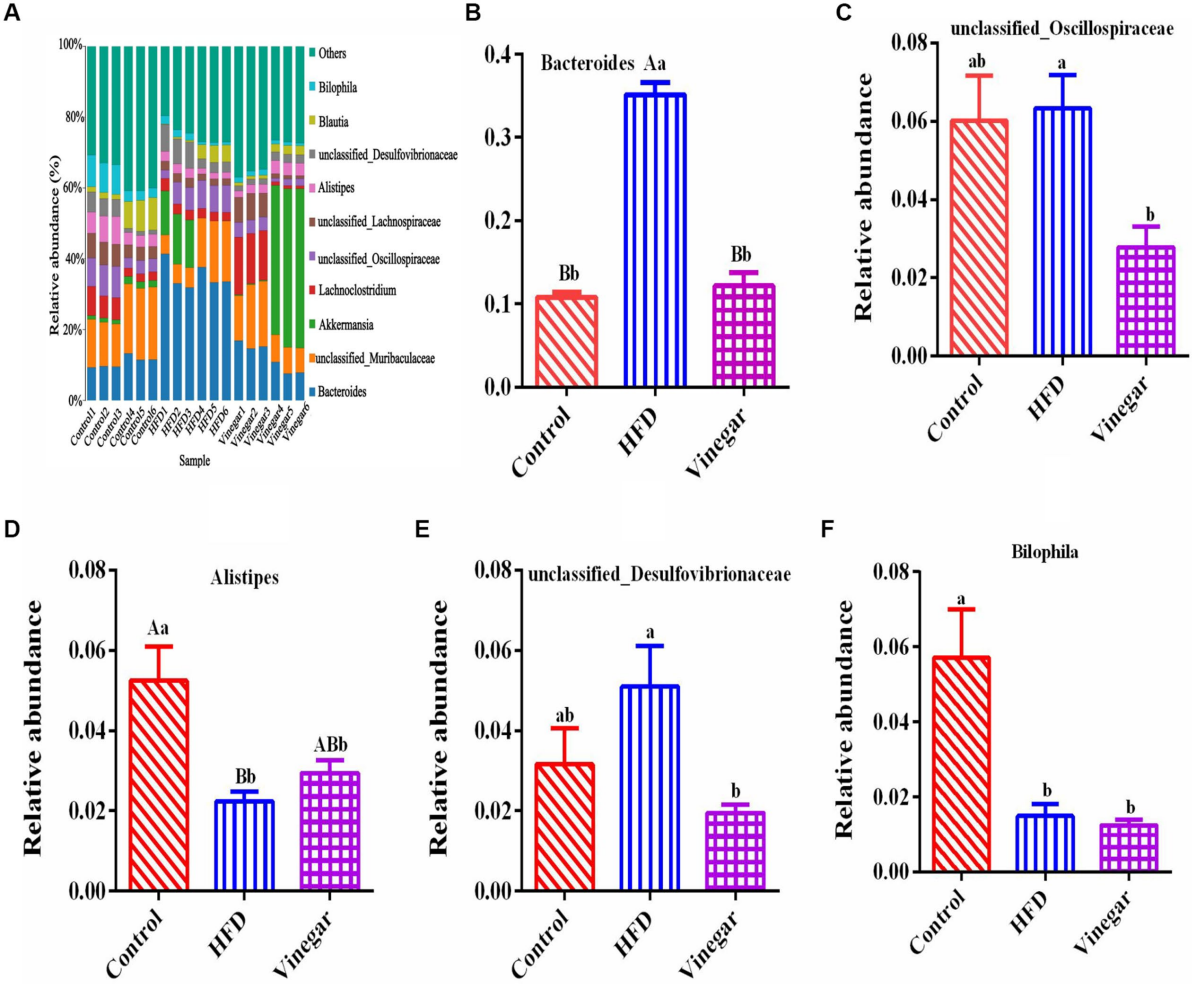
Relative abundances of gut microbiome in mice at the genus level. **(A)** A bar graph of genus distribution with a TOP 10 abundance, **(B)** relative abundance of *Bacteroidota* genus, **(C)** relative abundance of *unclassified_Oscillospiraceae* genus, **(D)** relative abundance of *Alistipes* genus, **(E)** relative abundance of *unclassified_Desulfovibrionaceae* genus, **(F)** relative abundance of *Bilophila* genus. Data are expressed as the means ± SEM, *n* = 6.

The distribution of the ten most abundant species (abundance ratio > 0.1%) was shown in Figure 6A, including *Bacteroides*, *Unclassified Muribaculaceae*, *Akkermansia, Lachnoclostridium*, *Unclassified_Oscillospiraceae*, *Unclassified_Lachnospiraceae*, *Alistipes*, *Unclassified_Desulfovibrionaceae*, *Blautia*, and *Bilophila*. Compared to the CON treatment, the relative abundance of the genus *Bacteroides* was significantly higher in the HFD treatment (Figure 6B, *p* < 0.01), as were the relative abundances of the *unclassified_Oscillospiraceae* and *the unclassified_Desulfovibrionaceae* (Figures 6C,E). Alistipes and Bilophila notably fell (Figures 6D, F, *p*<0.01, and *p*<0.05, respectively) in the HFD treatment. In contrast, compared to the HFD treatment. *Bacteroides* (Figure 6B, *p* < 0.01), *Unclassified_Oscillospiraceae* (Figure 6C, *p* < 0.05), and Unclassified_Desulfovibrionaceae (Figure 6E, *p* < 0.05) significantly decreased, Alistipes increased by 1.26 times (Figure 6D), and Bilophila decreased by 16.54% (Figure 6F) in the vinegar group.

**Figure 7:**
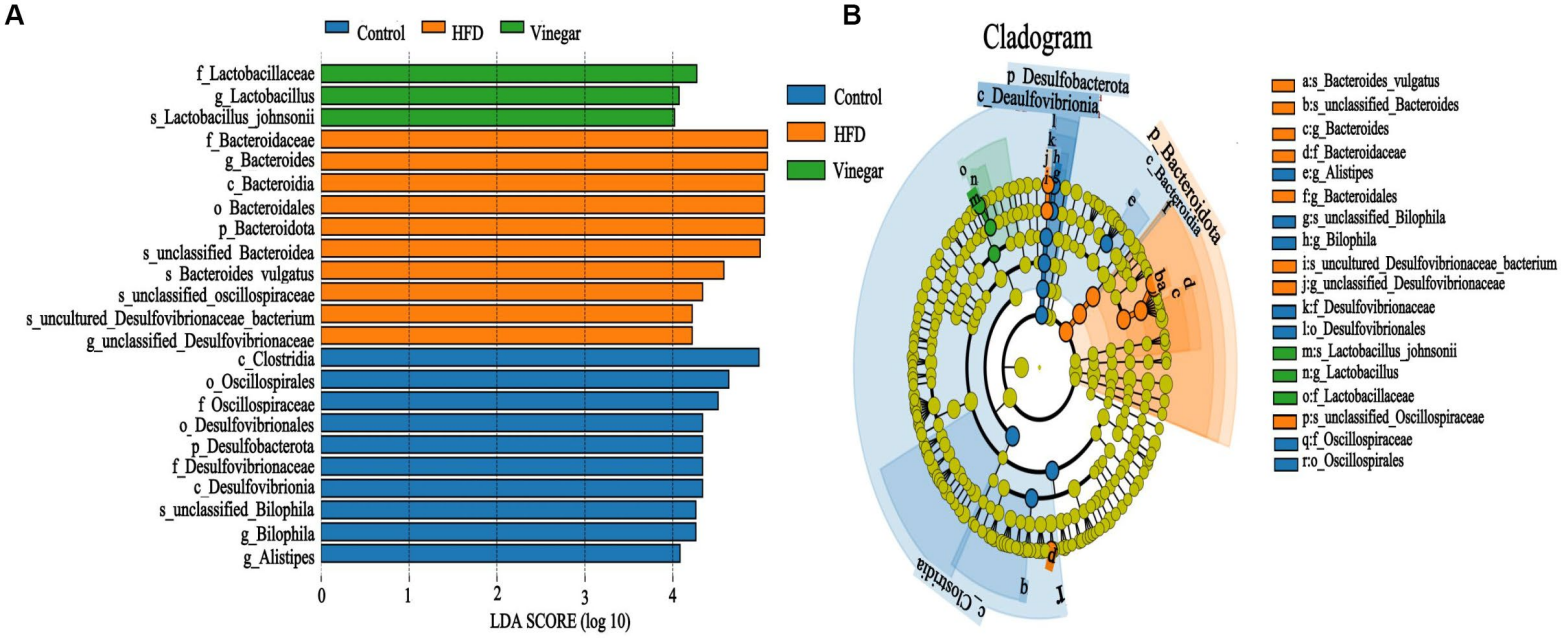
Lefse branch plot. **(A)** Histogram of distribution of LDA values reveal the microbiome of different taxa among the three groups. Panel **(B)** shows the different bacterial-rich taxa among the three groups. The concentric rings are species, genus, family, order, class, phylum, and so on. Blue, orange, and green show different bacterial taxa in the control, HFD and Vinegar groups, respectively, and yellow shows no significant differences between groups.

It is possible to identify biomarkers that statistically differ between groups using linear discriminant analysis (LDA). According to the LDA scores (Figure 7A) and the cladogram assay (Figure 7B), the representative gut microbes in the control treatment are the *Clostridia* class, *Oscillospirale*s, *Desulfovibrionales* orders, *Oscillospiraceae*, *Desulfovibrionaceae* families, *Bilophila*, and *Alistipes* genera. In contrast, *Bacteroidota* phylum, *Bacteroidia* class, *Bacteroidales*, *Desulfovibrionales* orders, *Bacteroidaceae*, *Oscillospiraceae*, *Desulfovibrionaceae* families, *Bacteroides*, and *Bacteroides vulgatus* genera were represented in the HFD treatment; *Lactobacillaceae* family and *Lactobacillus* genus were represented in the vinegar treatment.

### 3.6. Analysis of the correlation between gut microbiota and blood lipid indexes, antioxidant performances

**Figure 8:**
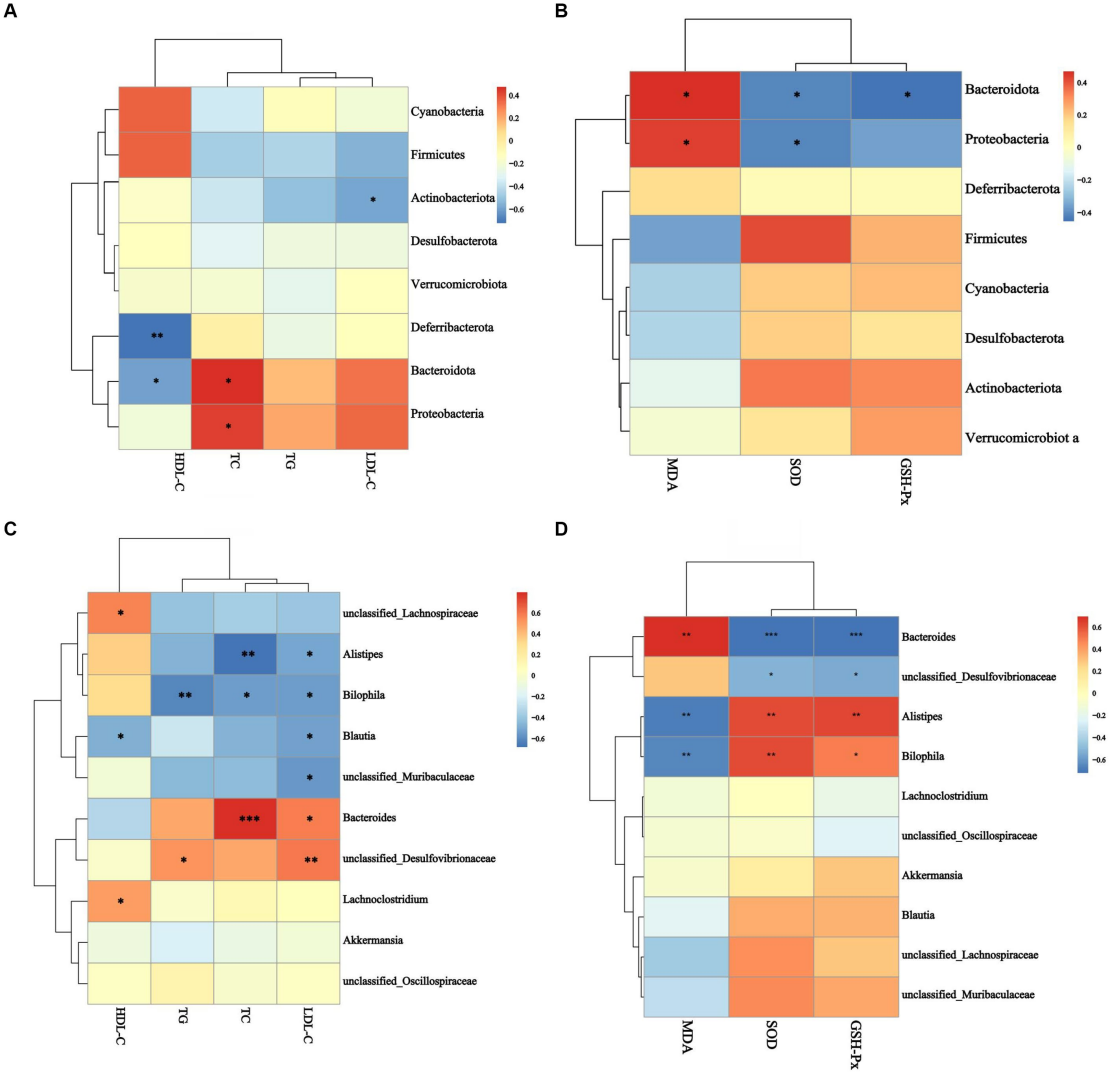
Analysis of the correlation between gut microbiota and blood lipid indexes antioxidant performance. **(A)** The correlation between gut microbiota and blood lipid index at phylum level, **(B)** the correlation between gut microbiota and blood lipid index at genus level, **(C)** the correlation between gut microbiota and antioxidant biomarker at phylum level, **(D)** the correlation between gut microbiota and antioxidant biomarker at genus level. ^*^p < 0.05, ^**^p < 0.05, ^***^p < 0.001.

Figures 8A,B showed the results of an analysis of the relationship between gut microbiomes and hyperlipidemia in mice. At the phylum level, *Bacteroidotas*, and *Proteobacteria* were positively correlated with TC (*p* < 0.05, Figure 8A). In contrast, *Bacteroidotas* (*p* < 0.05, Figure 8A), *Actinobacteriota* (*p* < 0.05, Figure 8A), and *Deferribacterota* (*p* < 0.01, Figure 8A) were negatively associated with HDL-C. At the genus level, *Bacteroides* were positively correlated with TC (*p* < 0.001, Figure 8B). A significant positive correlation between *Desulfovibrionaceae* and LDL-C was seen (*p* < 0.01, Figure 8B). *Bilophila* (*p* < 0.01, Figure 8B) demonstrated a significant negative correlation with TG, while *Unclassified_Muribaculaceae*, *Alistipes*, *Bilophila*, and *Blautia* all demonstrated a negative correlation with LDL-C (*p* < 0.01, Figure 8B). *Unclassified_Lachnospiraceae*, *Lachnoclostridium*, and *Blautia* showed a significant positive correlation with HDL-C (*p* < 0.05, Figure 8B), whereas Desulfovibrionaceae (*p* < 0.05, Figure 8B) was positively correlated with TG (*p* < 0.05, Figure 8B).

Figures 8C,D showed the correlation between gut microbiomes and antioxidant activity in mice. At the phylum level, *Bacteroidotas* and *Proteobacteria* were adversely correlated with serum SOD activity and positively correlated with serum MDA concentration (*p* < 0.05, Figure 8C). *Bacteroidotas* were associated negatively with serum GSH-Px activity (*p* < 0 0.05, Figure 8C). *Alistipes* and *Bilophila,* at the genus level, had positive correlations with serum SOD and GSH-Px activities and negative correlations with serum MDA concentration (*p* < 0.01, Figure 8D). *Bacteroides* were negatively correlated with serum SOD and GSH-Px activities (*p* < 0.001, Figure 8D) and positively correlated with serum MDA concentration (*p* < 0.01, Figure 8D). *Desulfovibrionaceae* was linked negatively with blood superoxide dismutase and glutathione peroxidase activities (*p* < 0.05, Figure 8D). These results showed that *Bacteroidotas*, *Proteobacteria*, *Desulfovibrionaceae*, and *Bacteroides* were inducers of hyperlipidemia and oxidative damage, and *Unclassified_Lachnospiraceae*, *Lachnoclostridium*, *Blautia*, *Alistipes,* and *Bilophila* were inhibitors of hyperlipidemia and oxidative damage.

### 3.7. BugBase phenotype prediction

**Figure 9:**
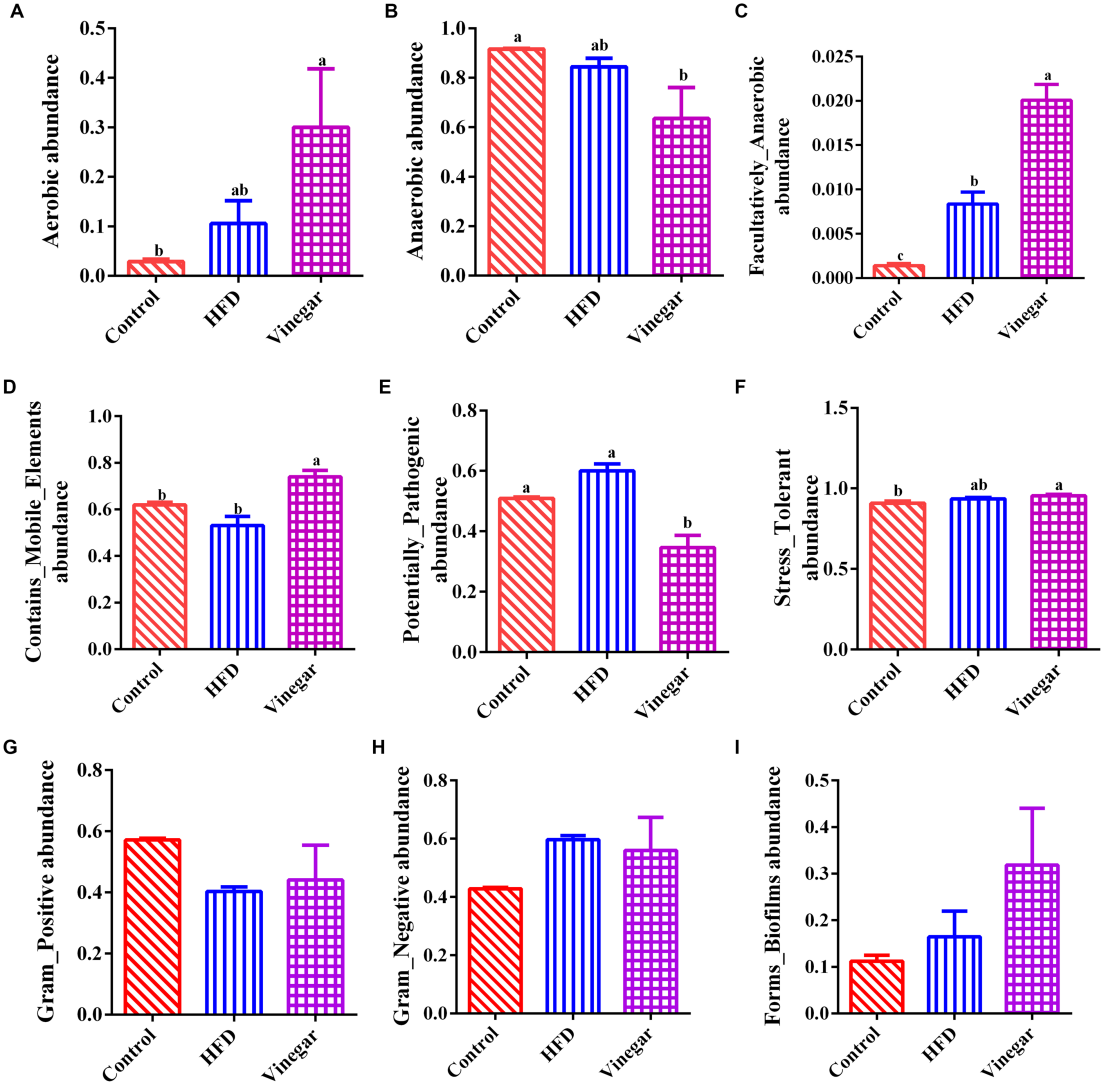
Prediction of BugBase phenotype. **(A)** Aerobic abundance, **(B)** Anaerobic abundance, **(C)** Facultatively_Anaerobic abundance, **(D)** Contains_Mobile_Elements abundance, **(E)** Potentially_pathogenic abundance, **(F)** Stress-tolerant abundance, **(G)** Gram_positive abundance, **(H)** Gram_negative abundance, **(I)** Forms_Biofilms abundance.

This study predicted nine potential phenotypes in the CON, HFD, and VIN treatments using Bugbase, and their relative abundances were compared (Supplementary Table 4). In the HFD treatment, compared to the CON treatment the Aerobic increased by 3.68 times, Anaerobic decreased by 9.07%, Contains_Mobile_Elements decreased by 16.14%, Facultative_Anaerobic increased significantly (*p* < 0.05, Figure 9D). Potential_Pathogens, Stress_Tolerant, Gram_Negative, Forms_Biofilms increased by 1.18, 1.03, 1.39 and 1.46 times, respectively (Figures 9E,F,H,I). Gram_Positive decreased by 29.55% (Figure 9G). In the VIN treatment, compared to the HFD treatment, Contains_Mobile_Elements and Facultative _Anaerobic were significantly higher (*p* < 0.05, Figures 9B–D), while Potential_Pathogens was considerably lower (*p* < 0.05, Figure 9E). Aerobic, Stress_Tolerant, Gram_Positive, Forms_Biofilms increased by 2.83, 1.02, 1.09, 1.94 times, respectively (Figures 9A,F,G,I).

**Figure 10:**
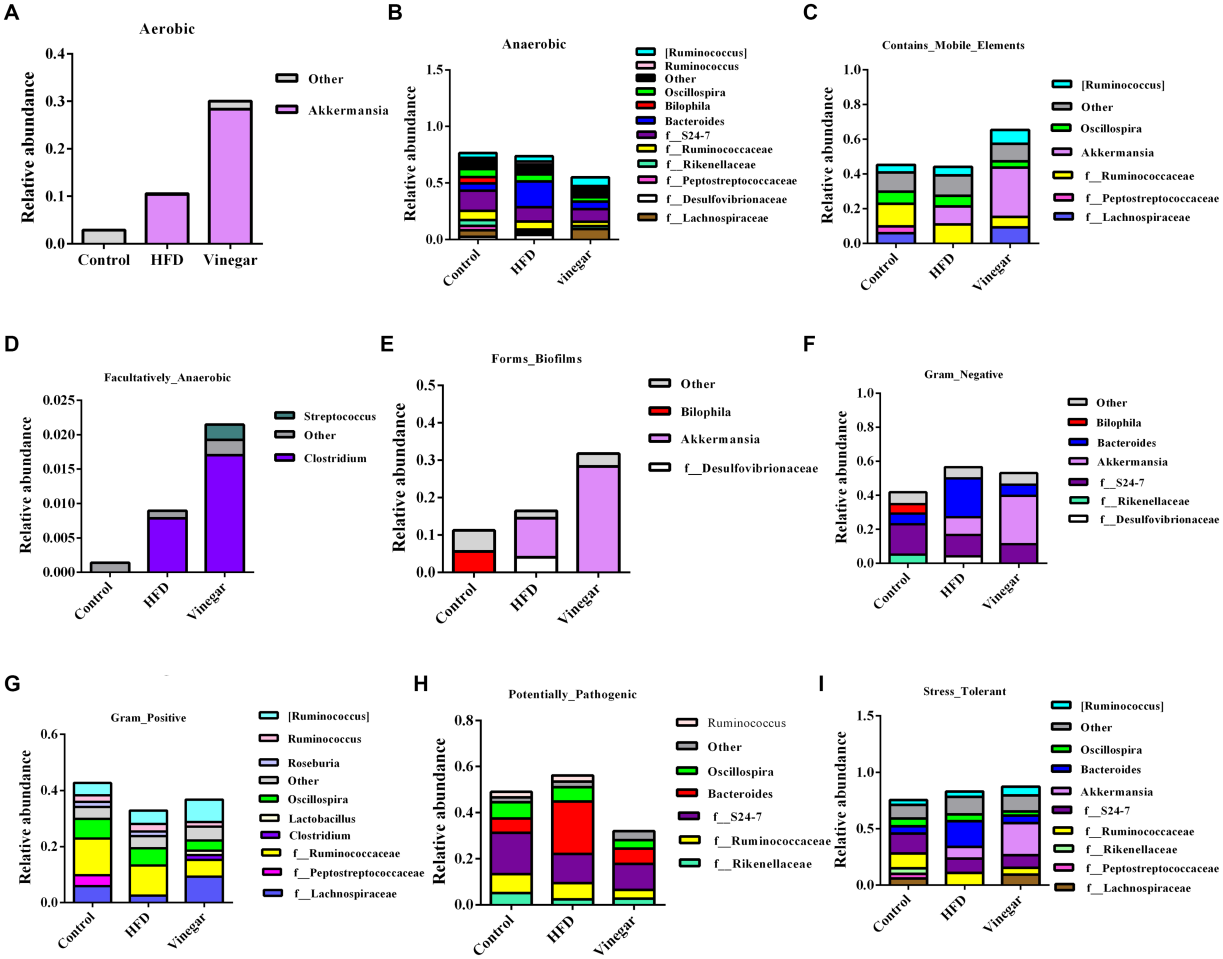
BugBase analysis of relative abundance of bacterial genera in mice. **(A)** Aerobic, **(B)** Anaerobic, **(C)** Contains_Mobile_Elements, **(D)** Facultatively_Anaerobic, **(E)** Forms_Biofilms, **(F)** Gram_Negative, **(G)** Gram_Positive, **(H)** Potential_Pathogens, **(I)** Stress_Tolerant.

The abundances of the nine phenotypes and the abundances of related genera were shown in Figure 10. The results suggested that HFD increased the abundances of Aerobic, Anaerobic, Facultative_anaerobic, Potential_Pathogens, Stress_Tolerant, and Contains_Mobile_Elements, Gram-Negative, and decreased the abundances of Contains_Mobile_Elements, Gram-Positive, which might be related to the increases of *Akkermansia, Enterobacteriaceae*, *Ruminococcus* and *Clostridium* and the decreases of *Bacteroides*, *Oscillospira*, *f_Ruminococcaceae*, *f_Rikenellaceae*, *f_24–7* (Supplementary Table 5); Aerobic, Anaerobic, Contains_Mobile_Elements, and Facultative_Anaerobic significantly increased than the HFD mice, and Potential_pathogens significantly decreased in the VIN treatment than in the HFD treatment, which may be related to the decreases of *Oscillospira*, *f_Peptostreptococcaceae*, *Bacteroides*, *f_Ruminococcaceae*, *f_Rikenellaceae* and the increases of *Akkermansia*, *f_Lachnospiraceae*, *Clostridium,* and *Streptococcus* (Supplementary Table 5) in the jujube vinegar group.

### 3.8. PICRUST2 function prediction

**Figure 11:**
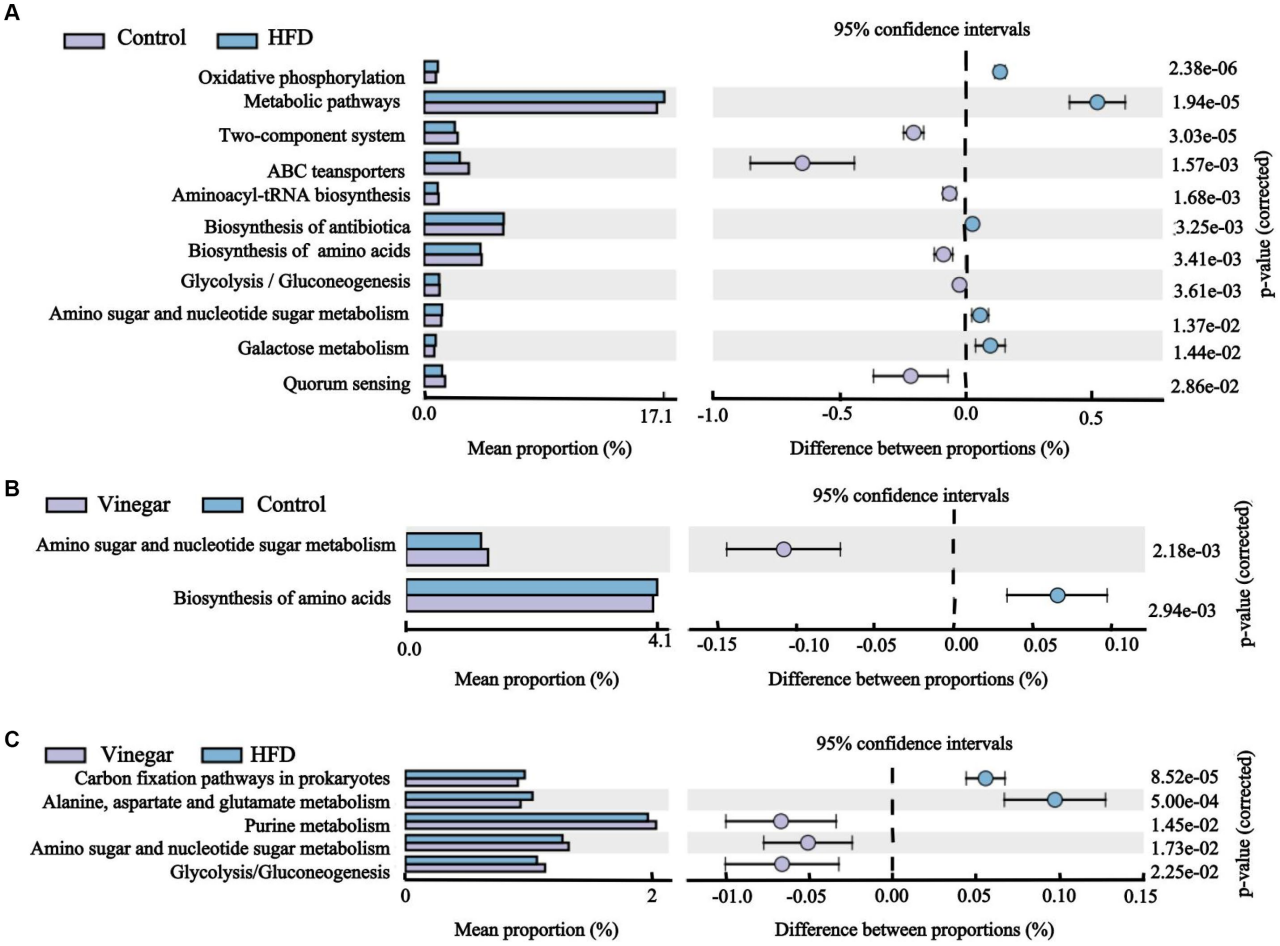
Histogram of KEGG pathway abundance and stamp analysis at level 3. **(A)** Stamp analysis between the control group and the HFD group, **(B)** Stamp analysis between the control group and the vinegar group, **(C)** Stamp analysis between the HFD group and the vinegar group.

We discovered 247 metabolic pathways at level 3 (Table 1). Compared to the CON treatment, seventy metabolic pathways were significantly upregulated in the HFD treatment including adipocytokine signaling pathway, fatty acid biosynthesis, fructose and mannose metabolism, metabolic pathways, PPAR signaling pathway, Type I diabetes mellitus, and so on, and 51 metabolic pathways were significantly downregulated in the HFD treatment, including ABC transport, glycerolipid metabolism, glycerophospholipid metabolism, HIF-1 signaling pathway, insulin resistance, insulin signaling pathway, and so on (Figure 11A; Supplementary Table 6), 27 metabolic pathways were significantly upregulated in the vinegar treatment, including D-Alanine metabolism, galactose metabolism, glucagon signaling pathway, glycolysis/gluconeogenesis, PPAR signaling pathway, phosphatidylinositol signaling system, and others; 38 metabolic pathways were significantly downregulated in the vinegar treatment, including amino acid biosynthesis, vancomycin group antibiotic biosynthesis, and fatty acid biosynthesis (Figure 11B; Supplementary Table 7). In comparison to the HFD treatment, 24 metabolic pathways were significantly upregulated in the vinegar treatment, including Alanine, aspartate, and glutamate metabolism, Biosynthesis of unsaturated fatty acids, Central carbon metabolism in cancer, D-Alanine metabolism, Glucagon signaling pathway, Glycolysis/Gluconeogenesis, Lysine biosynthesis, HIF-1 signaling pathway, and so on, while 45 metabolic pathways were significantly downregulated (Figure 11; Supplementary Table 8). The results showed that Metabolic pathways and HIF-1 signaling pathways at level 3 were somewhat improved in the vinegar treatment, implying that jujube vinegar may improve dyslipidemia *via* metabolic pathways associated with metabolic regulation and signal pathways associated with oxidative stress.

**Table 1:** Function composition table of KEEG at level 3.

Group	Control	HFD	Vinegar
Metabolic pathways	0.165	0.171	0.168
Biosynthesis of secondary metabolites	0.076	0.076	0.076
Biosynthesis of antibiotics	0.056	0.057	0.057
**Microbial metabolism in diverse**			
Environments	0.041	0.041	0.041
Biosynthesis of amino acids	0.041	0.040	0.040
ABC transporters	0.032	0.025	0.030
Carbon metabolism	0.027	0.027	0.027
Two-component system	0.024	0.022	0.022
Ribosome	0.023	0.022	0.023
Purine metabolism	0.0198	0.0197	0.0204
Other	0.495	0.497	0.501

## 4. Discussion

High blood triglyceride levels are associated with an increased chance of coronary artery disease, and TG is crucial for maintaining normal lipid metabolism (22, 23). HDL-C plays an important role in the transport of cholesterol and cholesteryl esters from tissues and cells to the liver, where they are metabolized to bile acids, and therefore, HDL-C has an essential function in reducing cholesterol levels in blood and peripheral tissues (23, 24). Conversely, LDL-C prevents cholesterol from breaking down in the liver and moving it to peripheral organs. Hyperlipidemia is characterized by increased serum TC, TG, and LDL-C and reduced HDL-C (24, 25). The usual method for estimating the efficacy of lipid-lowering medications is to look at the changes in serum TG, TC, and LDL-C levels and the rise in HDL-C levels (24, 26). According to some studies, pineapple vinegar, tomato vinegar, and persimmon vinegar may help improve metabolic syndrome, which is characterized by high cholesterol and triglyceride levels in serum samples brought on by high-fat diets and obesity (27–29). Fruit vinegar contains a variety of healthy ingredients, including several organic acids, minerals, carotenoids, and others, Studies showed that acetic acid inhibits the expression of lipogenic genes by activating AMPK, causing a reduction in the levels of fatty acid synthase and acetyl CoA carboxylase as a result (30). The present study showed that jujubes vinegar reduced serum LDL-C, TC, and TC/HDL-C in mice on a high-fat diet, consistent with Ali et al. (20). Hamden reported that date vinegar had antioxidant properties *in vitro* due to its high carotenoid content (21), Ali1reported that red and black date vinegar had antioxidant properties *in vitro* and contained phenols, flavonoids, and carotenoids (31). In this study, we found that jujube vinegar increased the activities of antioxidant enzymes, including SOD and GSH-Px, and decreased the MDA levels in the HFD mice. SOD and GSH-Px were generally regarded as the primary antioxidant enzyme defense system in animals and humans (32). SOD can catalyze superoxide into oxygen and hydrogen peroxide (H2O2) (33). GSH-Px catalyzes the conversion of reduced GSH to oxidized glutathione, protecting cells from disruption and damage caused by peroxide (34). MDA is the last product of lipid peroxidation, and it is an important indicator of body’s oxidative stress levels (35). The blood and hepatic indicators suggested that the jujube vinegar supplement could enhance the antioxidant ability in the HFD mice.

The gut microbiome carries several biological functions, such as regulation of the intestinal immune system axis, production of several essential metabolites, and support of good digestion through genes encoding digestive enzyme (36). The abundance and diversity of bacterial species in the human gut may indicate of health status (37–39). In this study, we used the Chao1, Ace, Shannon, and Simpson indexes to measure ɑ diversity, with Chao1 and Ace indexes measuring the numbers of species; and the Simpson and Shannon indexes measuring the abundance and homogeneity of species, the results found that jujube vinegar intervention could improve gut microbiome ɑ diversity in HFD mice, in agreement within agreement with the previous studies (39, 40). β diversity parameters were used to measure the distance among samples and similarity among the three groups and found that the control and vinegar treatments were far from the HFD treatment (Figures 4G,H), and significant clustering distribution in three treatments (Figure 4I), indicating the important role of jujube vinegar as a regulator of gut microbiome on HFD mice.

90% of the gut microbiome comprises the phyla *Firmicutes* and *Bacteroidota* (41). *Firmicutes* are gram-positive bacteria with rigid or semi-rigid cell walls, including *Bacillus*, *Clostridium*, *Enterococcus*, *Lactobacillus*, and *Ruminants*. *Bacteroidota* includes approximately 7,000 gram-negative species, mainly *Bacteroidetes*, *Mycobacteria*, *Bacteroidetes*, and *Prevotella* (42, 43). *Bacteroidota* have a high degree of functional redundancy, *Firmicutes* consist of a large number of functionally diverse core bacteria (44, 45). *Firmicutes* have been discovered to play a big part in modulating inflammation and preserving the intestinal barrier (46). Bacteria in *Bacteroidota* can release lipopolysaccharides, resulting in a higher inflammatory response (47); therefore, the decrease in *Phyllobacterium* spp. may be associated with lower inflammatory factors (48). The study of gut microbiome phylum levels showed that the abundance of *Bacteroidota* was significantly higher, the abundance of *Firmicutes* was significantly lower, and the F/B ratio was lower in the HFD treatment compared to the CON group. It is consistent with Wang and Gu et al.’s analysis (49, 50). The vinegar treatment showed a highly significant decrease in the abundance of *Bacteroidota*, a significant increase in the abundance of *Firmicutes*, and a decrease in the F/B ratio compared to the HFD treatment, in agreement with Mohamad’s study (51). The analysis of the genus level of gut microbiome revealed that the abundance of *Bacteroides* in the HFD treatment was 3.18 times higher than control treatment, and the abundance of *Bacteroides* in the vinegar treatment was 66.21% lower than the HFD group, in agreement with Li and Cristiane et al. (52, 53). the abundance of *unclassified_Oscillospiraceae* and *unclassified_Desulfovibrionaceae* was higher in the HFD treatment, *Desulfovibrionaceae* was considered to be one of the major endotoxin-producing pathogens (54), and in HFD mice, *Desulfovibrionaceae* (a harmful lipopolysaccharide-producing bacterium) exhibited a significant facilitative effect (52), *Desulfovibrionaceae* abundance decreased by 50.84% in the VIN treatment compared to the HFD treatment, in agreement with Li′s study (52). The abundance of the genus *Alistipes* in the HFD treatment fell by 54.41% compared to the CON treatment, in line with Fabersani et al.’s research (55). The abundance of *Alistipes* in the VIN treatment rose by 1.34-fold compared to the HFD treatment, *Alistipes* belongs to the family *Rikenellaceae* of *Mycobacterium*, which is a relatively new genus involved in colitis and regulation of colon cancer (56), and another study reported that abnormal parameters related to lipid metabolism in HFD mice were negatively correlated with the relative abundance of the genus *Alistipes*, suggesting a beneficial role of *Alistipes* (57). At LDA = 4.0, the marker genuses of the marker genuses in the VIN treatment was *Lactobacillu*s. The results suggested that jujube vinegar regulated gut microbiota structure in mice fed a high-fat diet by inhibiting the abundance of harmful bacteria such as *Desulfovibrionaceae*, *Bacteroides*, and increasing the abundance of beneficial bacteria such as *Lactobacillus, Alistipes*, consistent with the results of dominant genera at the genus level.

We analyzed further the relationships of gut microbiota and TC, TG, LDL-C, HDL-C, MDA, SOD, and GSH-Px. The results of the correlation suggested that *Alistipes* and *Bilophila* may play a role in improving lipid metabolism and enhancing antioxidant capacity; *Blautia* and *unclassified_Muribaculacea*e may play a role in improving lipid metabolism. In contrast, *Bacteroidota*, *Proteobacteria*, *Bacteroides, and unclassified_Desulfovibrionaceae* caused the disorder of lipid metabolism and oxidative stress in the HFD mice, similar to the studies of Li and Yu et al. (58, 59).

The abundances of Aerobic, contains_Mobile_Elements, Facultatively_Anaerobic were higher in the VIN treatment than in the HFD treatment this may be related to the decreases of *Oscillospira*, *f_Peptostreptococcaceae*, *Bacteroides*, *f_Ruminococcaceae*, and *f_Rikenellaceae*, and the increases of *Akkermansia*, *f_Lachnospiraceae*, *Clostridium*, and *Streptococcus* induced by jujube vinegar in high-fat mice. *Peptostreptococcaceae* plays a role in atherosclerosis (60). The relative abundance of *Ruminococcaceae* decreased in the vinegar group, consistent with Li et al. (61). *Akkermansia* is a Gram-negative bacterium belonging to the *Verrucomicrobacteria*, which produces mucin-degrading enzymes that ferment mucin to acetate, propionate and sulfate (62), according to clinical and preclinical studies, *Akkermansia* was found to be negatively associated with metabolic disorders (63–65).

We predicted the target genes of high-fat diet and jujube vinegar to understand better how they work. According to KEGG pathway analysis, a high-fat diet primarily enriched the signaling pathways, for example, apoptosis, phospholipase D signaling, adipocytokine signaling, and lipopolysaccharide biosynthesis. It was important to note that high-fat diet were also involved in the enrichment of the PPAR signaling pathway, fatty acid biosynthesis, and the downregulation of glycolipid metabolism, glycerophospholipid metabolism, and other pathways related to obesity and fat deposition, which can facilitate the development of adipocytes and lipid accumulation (66); High-fat diet downregulated glycolysis/gluconeogenesis, insulin resistance, and upregulated fructose and mannose metabolism, galactose metabolism, citrate cycle (TCA cycle), and other pathways related to blood glucose (66); in addition, high-fat diet upregulated oxidative phosphorylation, peroxisome, and glutathione metabolism which caused oxidative stress (67), with the consistent with our previous study Figure 3. Interestingly, high-fat diet downregulated HIF-1 signaling pathway, which regulated glucose catabolism and energy metabolism, Fatty acid synthesis, ROS levels (68), and upregulated novobiocin biosynthesis, Cationic antimicrobial peptide (CAMP) resistance, monobactam biosynthesis, pathogenic *Escherichia coli* infection, shigellosis, and vibrio cholerae infection. On the other hand, the result of the VIN treatment showed an enrichment in the biosynthesis of unsaturated fatty acids, the glucagon signaling pathway, glycolysis/gluconeogenesis, purine metabolism, fatty acid biosynthesis, and other pathways in HFD mice, and a downregulation of the amino sugar and nucleotide sugar metabolism, the arachidonic acid metabolism, the steroid hormone biosynthesis, and other pathways, as well as the peroxisome and the PPAR signaling pathway. It was interesting to note that jujube vinegar downregulated cationic antimicrobial peptide (CAMP) resistance while upregulating the biosynthesis of antibiotics like neomycin, kanamycin, gentamicin, and streptomycin, which are produced by the fermentation of actinobacterial Streptomyces (69, 70), consistent with the results of dominant phylum (Supplementary Table 2). However, due to the limitations of the sample size and animal models, the present findings can only provide some reference for exploring the mechanisms of jujube vinegar on HFD-induced mice’s dyslipidemia, which needs to be validated by studies with large sample sizes.

## 5. Conclusion

Jujube vinegar may modify microbial diversity, structure, and function to improve hyperlipoidemia induced by high-fat diet. It is also possible to improve hyperlipoidemia-related liver lesions and antioxidant performance by regulating intestinal related metabolic pathways. These results strongly suggest that jujube vinegar may alleviate hyperlipoidemia and oxidative damage by preventing gut microbiome disorder. Moreover, jujube vinegar can treat hyperlipoidemia with multiple components, multiple metabolic pathways, and multiple targets through gut microbiome. This research offers a fresh perspective on the role of jujube vinegar in hyperlipoidemia and suggests that jujube vinegar acts as a preventative measure.

## Data availability statement

The original contributions presented in the study are included in the article/Supplementary material, further inquiries can be directed to the corresponding author.

## Ethics statement

The animal study was reviewed and approved by Agriculture University Institutional Animal Care and Use Committee of Shanxi Agriculture University. Written informed consent was obtained from the owners for the participation of their animals in this study.

## Author contributions

LL designed and performed all experiments, data curation, and writing original draft. GD designed all experiments, funding acquisition, data curation, and writing—review and editing. All authors contributed to the article and approved the submitted version.

## Funding

This research was funded by the Key R&D projects of Shanxi Province, grant no. 201703D221028-1.

## Conflict of interest

The authors declare that the research was conducted in the absence of any commercial or financial relationships that could be construed as a potential conflict of interest.

## Publisher’s note

All claims expressed in this article are solely those of the authors and do not necessarily represent those of their affiliated organizations, or those of the publisher, the editors and the reviewers. Any product that may be evaluated in this article, or claim that may be made by its manufacturer, is not guaranteed or endorsed by the publisher.

## Footnotes

^1^https://dataview.ncbi.nlm.nih.gov/object/PRJNA934872?reviewerrpieibr5rs7hd8jl5hgmot99lt

^2^www.biocloud.ent
