# Supplementary material for: Deciphering the mechanism of jujube vinegar on hyperlipoidemia through gut microbiome based on 16S rRNA, BugBase analysis, and the stamp analysis of KEEG
Source: Front Nutr. 2023 May 19;10:1160069. doi: 10.3389/fnut.2023.1160069 (PMC10235701; doi:10.3389/fnut.2023.1160069)
Supplement: Supplementary file 1 [file Data_Sheet_1.zip › TableS1.docx]

**x** ±±

**Supplementary table 1 jujube vinegar components(** )

| Ingredients Amount (%) Ingredients unit Amount |
| --- |
| acid 39.5 pH 2.82±0.07  alcohols 10.48 conductivity mS/cm 3.98±0.09  esters 34.13 amino acids mg/100mL 5.04±0.06  phenols 0.03 total sugar content % 1.82±0.12  aldehydes 11.37 fat content g/100mL 0.66±0.17  heterocyclic class 0.02 protein content g/100mL 1.33±0.20  Others 2.72 total titratable acidity w/v 3.81±0.13 |
