# Supplementary material for: Deciphering the mechanism of jujube vinegar on hyperlipoidemia through gut microbiome based on 16S rRNA, BugBase analysis, and the stamp analysis of KEEG
Source: Front Nutr. 2023 May 19;10:1160069. doi: 10.3389/fnut.2023.1160069 (PMC10235701; doi:10.3389/fnut.2023.1160069)
Supplement: Supplementary file 1 [file Data_Sheet_1.zip › TableS2.docx]

**Supplementary table 2 Sample sequencing data (**  ±SEM)

**x** ±

| Raw Clean Denoised Merged Non-chimeric  Reads reads Reads Reads Reads |
| --- |
| the control group 58398±2446 41624±1660 41029±1630 35165±1917 30452±1943  the HFD group 65572±6351 46757±1502 46329±1476 42122±1618 38104±1373  the vinegar group 61833±2942 43116±2121 42735±2121 40131±2259 38977±2220 |
