# Supplementary material for: Deciphering the mechanism of jujube vinegar on hyperlipoidemia through gut microbiome based on 16S rRNA, BugBase analysis, and the stamp analysis of KEEG
Source: Front Nutr. 2023 May 19;10:1160069. doi: 10.3389/fnut.2023.1160069 (PMC10235701; doi:10.3389/fnut.2023.1160069)
Supplement: Supplementary file 1 [file Data_Sheet_1.zip › TableS3.docx]

| the control group the HFD group the vinegar group |
| --- |
| Firmicutes 0.51±0.003 0.32±0.006 0.41±0.035  Bacteroidota 0.37±0.008 0.54±0.014 0.32±0.008  Verrucomicrobiota 0.015±0.0007 0.066±0.012 0.22±0.04  Desulfobacterota 0.089±0.009 0.066±0.005 0.032±0.0004  Cyanobacteria 0.001±0.00007 0.001±0.0004 0.016±0.0027  Deferribacterota 0.011±0.0013 0.004±0.0004 0.00003±0.000008  Actinobacteriota 0.006±0.0009 0.002±0.0003 0.004±0.0005  Proteobacteria 0.0004±0.00004 0.002±0.00003 0.0015±0.00017 |

**x** ±±±

Supplementary table 3 the abundances at the phylum level ( ± SEM)
