# Supplementary material for: Deciphering the mechanism of jujube vinegar on hyperlipoidemia through gut microbiome based on 16S rRNA, BugBase analysis, and the stamp analysis of KEEG
Source: Front Nutr. 2023 May 19;10:1160069. doi: 10.3389/fnut.2023.1160069 (PMC10235701; doi:10.3389/fnut.2023.1160069)
Supplement: Supplementary file 1 [file Data_Sheet_1.zip › TableS4.docx]

**Supplementary table 4 The nine potential phenotypes abundances( ±SEM)**

**x**

| potential phenotypes the control group the HFD group the vinegar goup |
| --- |
| Aerobic 0.028±0.002^b^ 0.106±0.001^ab^ 0.300±0.048^a^  Anaerobic 0.919±0.001^a^ 0.842±0.014^ab^ 0.632±0.052^b^  Contains_Mobile_Elements 0.600±0.001^b^ 0.517±0.014^b^ 0.748±0.009^a^  Forms_Biofilm 0.112±0.005 0.164±0.023 0.318±0.050  Gram_positive 0.572±0.002 0.403±0.006 0.441±0.046  Gram_negative 0.428±0.002 0.597±0.006 0.559±0.046  Potentially_pathogenic 0.499±0.004^a^ 0.592±0.010^a^ 0.327±0.014^b^  Stress_tolerant 0.908±0.006^b^ 0.935±0.004^ab^ 0.955±0.003^a^  Facultatively_Anaerobic 0.001±0.0001^c^  0.009±0.0006^b^ 0.021±0.0007^a^ |

.
