# Supplementary material for: Deciphering the mechanism of jujube vinegar on hyperlipoidemia through gut microbiome based on 16S rRNA, BugBase analysis, and the stamp analysis of KEEG
Source: Front Nutr. 2023 May 19;10:1160069. doi: 10.3389/fnut.2023.1160069 (PMC10235701; doi:10.3389/fnut.2023.1160069)
Supplement: Supplementary file 1 [file Data_Sheet_1.zip › TableS5.docx]

**Supplementary table 5 The species abundances of nine potential phenotypes**

**at genus level**

| potential phenotypes species the control group the HFD group the vinegar goup |
| --- |
| Aerobic Akkermansia 0 0.104 0.284  Anaerobic f__Desulfovibrionaceae 0.023 0.041 0  f__Lachnospiraceae 0.059 0.025 0.093  f__Peptostreptococcaceae 0.039 0 0  f__Rikenellaceae 0.051 0.024 0.026  f__Ruminococcaceae 0.082 0.071 0.038  f__S24-7 0.179 0.126 0.113  Bacteroides 0.063 0.229 0.066  Bilophila 0.056 0 0  Oscillospira 0.070 0.061 0.037  Ruminococcus 0.044 0.047 0.079  Contains_Mobile_Elements f__Lachnospiraceae 0.059 0 0.093  f__Peptostreptococcaceae 0.039 0 0  f__Ruminococcacea 0.131 0.108 0.060  Akkermansia 0 0.104 0.284  Oscillospira 0.070 0.061 0.037  Forms_Biofilm f__Desulfovibrionaceae 0 0.041 0  Akkermansia 0 0.104 0.284  Bilophila 0.056 0 0  Gram_positive f__Lachnospiraceae 0.059 0.025 0.093  f__Peptostreptococcaceae 0.039 0 0  f__Ruminococcaceae 0.131 0.128 0.60  Clostridium 0 0 0.017  Lactobacillus 0 0 0.015  Oscillospira 0.070 0.061 0.037  Roseburia 0.019 0.017 0  Ruminococcus 0.044 0.047 0.079  Gram_negative f__Desulfovibrionaceae 0 0.041 0  f__Rikenellaceae 0.051 0 0  Akkermansia 0 0.104 0.284  Bacteroides 0.063 0.229 0.066  Bilophila 0.056 0 0  Potentially_pathogenic f__Rikenellaceae 0.051 0.024 0.026  f__Ruminococcaceae 0.082 0.071 0.038  f__S24-7 0.179 0.126 0.113  Bacteroides 0.063 0.229 0.066  Oscillospira 0.070 0.061 0.037  Ruminococcus 0.023 0.027 0  Stress_tolerant f__Lachnospiraceae 0.059 0 0.093  f__Peptostreptococcaceae 0.039 0 0  f__Rikenellaceae 0.051 0 0  f__Ruminococcaceae 0.131 0.128 0.60  f__S24-7 0.179 0.126 0.113  Akkermansia 0 0.104 0.284  Bacteroides 0.063 0.229 0.066  Oscillospira 0.070 0.061 0.037  Ruminococcus 0.044 0.047 0.079  Facultatively_Anaerobic Clostridium 0 0.008 0.017  Streptococcus 0 0 0.0022 |
