# Supplementary material for: Deciphering the mechanism of jujube vinegar on hyperlipoidemia through gut microbiome based on 16S rRNA, BugBase analysis, and the stamp analysis of KEEG
Source: Front Nutr. 2023 May 19;10:1160069. doi: 10.3389/fnut.2023.1160069 (PMC10235701; doi:10.3389/fnut.2023.1160069)
Supplement: Supplementary file 1 [file Data_Sheet_1.zip › TableS6.docx]

**Supplementary table 6 Stamp analysis of the abundances in metabolic pathways between the contrl group and the HFD group at level 3 (** ± std **)**

**x**

| metabolic pathways the control group the HFD group *P* |
| --- |
| ABC transporters 3.17±0.17 2.53±0.06 0.0003  Adipocytokine signaling pathway 0.07±0.0009 0.09±0.004 0.046  Alcoholism 0.00001±0.000005 0.000003±0.000004 0.01  Apoptosis 0.02±0.003 0.04±0.002 0.00009  Bacterial chemotaxis 0.60±0.02 0.40±0.03 0.0000003  Bacterial invasion of epithelial cells 0.0000271±0.00004 0.0003±0.0002 0.00002  Biosynthesis of amino acids 4.086±0.01 3.999±0.03 0.0009  Biosynthesis of antibiotics 5.63±0.0099 5.65±0.005 0.0007  Biosynthesis of unsaturated fatty acids 0.11±0.008 0.10±0.004 0.02  Cationic antimicrobial peptide (CAMP) resistance 0.26±0.03 0.33±0.01 0.001  Citrate cycle (TCA cycle) 0.61±0.02 0.65±0.01 0.001   1. Alanine metabolism 0.096±0.004 0.088±0.002 0.008   Drug metabolism - cytochrome P450 0.0188±0.001 0.025±0.004 0.01  Fatty acid biosynthesis 0.519±0.004 0.546±0.01 0.002  Fructose and mannose metabolism 0.722±0.013 0.753±0.018 0.014  Galactose metabolism 0.723±0.05 0.821±0.02 0.005  Glutathione metabolism 0.128±0.013 0.169±0.006 0.0003  Glycerolipid metabolism 0.333±0.005 0.274±0.007 0.000000126  Glycerophospholipid metabolism 0.482±0.002 0.450±0.008 0.0001  Glycolysis / Gluconeogenesis 1.093±0.009 1.069±0.006 0.0009  HIF-1 signaling pathway 0.101±0.0006 0.0942±0.001 0.00000603  Insulin resistance 0.1005±0.004 0.0911±0.005 0.007  Insulin signaling pathway 0.0722±0.004 0.0655±0.005 0.04  Lipopolysaccharide biosynthesis 0.227±0.003 0.285±0.015 0.0002  Metabolic pathways 16.54±0.085 17.07±0.069 0.00000125  Metabolism of xenobiotics by cytochrome P450 0.0188±0.001 0.0254±0.004 0.01  Mineral absorption 0.00527±0.001 0.0036±0.001 0.0046  Novobiocin biosynthesis 0.133±0.003 0.138±0.002 0.02  Oxidative phosphorylation 0.837±0.016 0.974±0.013 0.0000000767  PPAR signaling pathway 0.121±0.002 0.148±0.005 0.00000658  Pathogenic Escherichia coli infection 0.00000176±0.0000025 0.0000227±0.0000126 0.01  Pentose and glucuronate interconversions 0.396±0.021 0.461±0.016 0.000367  Pentose phosphate pathway 0.806±0.017 0.783±0.007 0.025  Phosphatidylinositol signaling system 0.0797±0.001 0.0893±0.003 0.0006  Phospholipase D signaling pathway 0.0172±0.0009 0.0230±0.002 0.0008  Phosphotransferase system (PTS) 0.193±0.03 0.124±0.01 0.007  Protein digestion and absorption 0.022±0.005 0.050±0.003 0.00000374  Protein processing in endoplasmic reticulum 0.0745±0.002 0.0793±0.002 0.01  Synthesis and degradation of ketone bodies 0.0412±0.006 0.0276±0.007 0.01  Type I diabetes mellitus 0.0522±0.003 0.0427±0.0006 0.004  Vitamin B6 metabolism 0.163±0.0009 0.180±0.002 0.0000012  beta-Alanine metabolism 0.0894±0.006 0.0974±0.0005 0.03 |
