# Supplementary material for: Deciphering the mechanism of jujube vinegar on hyperlipoidemia through gut microbiome based on 16S rRNA, BugBase analysis, and the stamp analysis of KEEG
Source: Front Nutr. 2023 May 19;10:1160069. doi: 10.3389/fnut.2023.1160069 (PMC10235701; doi:10.3389/fnut.2023.1160069)
Supplement: Supplementary file 1 [file Data_Sheet_1.zip › TableS7.DOCX]

**Supplementary table 7 Stamp analysis of the abundances in metabolic pathways between the control group and the vinegar group ( ± std )**

**x**

| metabolic pathways the control group the vinegar group *P* |
| --- |
| Bacterial chemotaxis 0.60±0.02 0.422±0.08 0.004  Bacterial invasion of epithelial cells 0.0000271±0.00004 0.000273±0.0002 0.00002  Biosynthesis of amino acids 4.086±0.01 4.021±0.03 0.001  Biosynthesis of vancomycin group antibiotics 0.0480±0.005 0.0386±0.0004 0.01   1. Alanine metabolism 0.096±0.004 0.103±0.002 0.008   Fatty acid biosynthesis 0.519±0.004 0.499±0.004 0.00002  Fatty acid metabolism 0.536±0.016 0.505±0.02 0.03  Galactose metabolism 0.723±0.05 0.818±0.116 0.009  Glucagon signaling pathway 0.142±0.009 0.159±0.007 0.008  Glycolysis / Gluconeogenesis 1.093±0.03 1.135±0.03 0.02  Mineral absorption 0.00527±0.00122 0.002±0.0003 0.001  PPAR signaling pathway 0.121±0.002 0.125±0.003 0.0492  Phosphatidylinositol signaling system 0.0797±0.001 0.0829±0.002 0.015  Phospholipase D signaling pathway 0.0172±0.0009 0.0144±0.00096 0.00095  Vitamin B6 metabolism 0.163±0.0009 0.167±0.002 0.001 |
