# Supplementary material for: Deciphering the mechanism of jujube vinegar on hyperlipoidemia through gut microbiome based on 16S rRNA, BugBase analysis, and the stamp analysis of KEEG
Source: Front Nutr. 2023 May 19;10:1160069. doi: 10.3389/fnut.2023.1160069 (PMC10235701; doi:10.3389/fnut.2023.1160069)
Supplement: Supplementary file 1 [file Data_Sheet_1.zip › TableS8.docx]

**Supplementary table 8 Stamp analysis of the abundances in metabolic pathways between the HFD group and thevinegar group (** ± std **)**

**x**

| metabolic pathways the HFD group the vinegar group *P* |
| --- |
| Adipocytokine signaling pathway 0.09±0.004 0.0686±0.003 0.00000341  Apoptosis 0.0384±0.003 0.0240±0.004 0.0000715  Biosynthesis of unsaturated fatty acids 0.101±0.004 0.120±0.005 0.0000904  Biosynthesis of vancomycin group antibiotics 0.0392±0.0013 0.0480±0.005 0.012  Cationic antimicrobial peptide (CAMP) resistance 0.327±0.0108 0.263±0.03 0.004   1. Alanine metabolism 0.088±0.002 0.103±0.002 0.00000138   D-Glutamine and D-glutamate metabolism 0.181±0.006 0.172±0.005 0.03  Drug metabolism - cytochrome P450 0.025±0.004 0.0184±0.004 0.03  Fatty acid biosynthesis 0.545±0.01 0.500±0.004 0.0000723  Fatty acid metabolism 0.544±0.0186 0.505±0.021 0.01  Glucagon signaling pathway 0.134±0.004 0.159±0.007 04 0.0000624  Glutathione metabolism 0.169±0.006 0.135±0.006 0.0000044  Glycerolipid metabolism 0.274±0.007 0.334±0.05 0.05  Glycerophospholipid metabolism 0.450±0.007 0.479±0.02 0.03  Glycolysis / Gluconeogenesis 1.069±0.006 1.135±0.0296 0.004  HIF-1 signaling pathway 0.094±0.001 0.107±0.01 0.05  Insulin resistance 0.091±0.005 0.091±0.004 0.04  Insulin signaling pathway 0.065±0.005 0.075±0.008 0.045  Metabolism of xenobiotics by cytochrome P450 0.025±0.004 0.018±0.004 0.02  Mineral absorption 0.0036±0.001 0.002±0.0003 0.03  PPAR signaling pathway 0.148±0.005 0.125±0.003 0.0000145  Phosphatidylinositol signaling system 0.0893±0.003 0.0829±0.002 0.004  Phospholipase D signaling pathway 0.023±0.002 0.0144±0.00096 0.0000716  Protein digestion and absorption 0.05±0.003 0.025±0.007 0.0000855  Protein processing in endoplasmic reticulum 0.0793±0.002 0.067±0.007 0.009  Vitamin B6 metabolism 0.180±0.002 0.167±0.002 0.00000225 |
